# Validation of a Computerized, Game-based Assessment Strategy to Measure Training Effects on Motor-Cognitive Functions in People With Dementia

**DOI:** 10.2196/games.5696

**Published:** 2016-07-18

**Authors:** Stefanie Wiloth, Nele Lemke, Christian Werner, Klaus Hauer

**Affiliations:** ^1^ AGAPLESION Bethanien Hospital, Geriatric Centre of the University of Heidelberg Heidelberg Germany; ^2^ The Institute for the Study of Christian Social Service at the University of Heidelberg Heidelberg Germany; ^3^ Network of Aging Research (NAR), University of Heidelberg Heidelberg Germany

**Keywords:** serious games, computerized assessment, validation, motor-cognitive functions, elderly, older adults, cognitive impairment, dementia

## Abstract

**Background:**

Exergames often used for training purpose can also be applied to create assessments based on quantitative data derived from the game. A number of studies relate to these use functionalities developing specific assessment tasks by using the game software and provided good data on psychometric properties. However, (1) assessments often include tasks other than the original game task used for training and therefore relate to similar but not to identical or integrated performances trained, (2) people with diagnosed dementia have insufficiently been addressed in validation studies, and (3) studies did commonly not present validation data such as sensitivity to change, although this is a paramount objective for validation to evaluate responsiveness in intervention studies.

**Objective:**

Specific assessment parameters have been developed using quantitative data directly derived from the data stream during the game task of a training device (Physiomat). The aim of this study was to present data on construct validity, test–retest reliability, sensitivity to change, and feasibility of this internal assessment approach, which allows the quantification of Physiomat training effects on motor-cognitive functions in 105 multimorbid patients with mild-to-moderate dementia (mean age 82.7±5.9).

**Methods:**

Physiomat assessment includes various tasks at different complexity levels demanding balance and cognitive abilities. For construct validity, motor-cognitive Physiomat assessment tasks were compared with established motor and cognitive tests using Spearman’s rank correlations (r_s_). For test–retest reliability, we used intra-class correlations (ICC_3,1_) and focused on all Physiomat tasks. Sensitivity to change of trained Physiomat tasks was tested using Wilcoxon statistic and standardized response means (SRMs). Completion rate and time were calculated for feasibility.

**Results:**

Analyses have mostly shown moderate-to-high correlations between established motor as well as cognitive tests and simple (r_s_=−.22 to .68, *P* ≤.001-.03), moderate (r_s_=−.33 to .71, *P* ≤.001-.004), and complex motor-cognitive Physiomat tasks (r_s_=−.22 to .83, *P* ≤.001-.30) indicating a good construct validity. Moderate-to-high correlations between test and retest assessments were found for simple, moderate, and complex motor-cognitive tasks (ICC=.47-.83, *P* ≤.001) indicating good test–retest reliability. Sensitivity to change was good to excellent for Physiomat assessment as it reproduced significant improvements (*P* ≤.001) with mostly moderate-to-large effect sizes (SRM=0.5-2.0) regarding all trained tasks. Completion time averaged 25.8 minutes. Completion rate was high for initial Physiomat measures. No adverse events occurred during assessment.

**Conclusions:**

Overall, Physiomat proved to have good psychometric qualities in people with mild-to-moderate dementia representing a reliable, valid, responsive, and feasible assessment strategy for multimorbid older adults with or without cognitive impairment, which relates to identical and integrated performances trained by using the game.

## Introduction

With respect to the growing elderly population, the role of innovative assessments to detect motor-functional or cognitive deficits is becoming increasingly important. Such assessment methods could help to identify appropriate interventions to delay age-related physical or mental decline.

### Using Exergames for Training and Assessment Purpose

Modern computer technology has yielded creative and motivating procedures especially for training mental and physical abilities, thereby reducing fall risk in older adults and promoting independence and participation in everyday life, respectively. Recent studies could show that applications such as tablet mobile devices with integrated memory training apps can be used even by people with early stage of dementia [1,2]. Some reviews reported that exergames that combine physical activity with digital gaming have also been found wide application in healthy [3], disabled older adults [4], and in people with cognitive impairment or dementia [5-7]. Just to name a few examples, a computerized Tai Chi game using the Microsoft’s motion-capture device Kinect [8] or a computer tele-rehabilitation platform that combines game-based exercises with telemonitoring [9] has the potential to improve motor and cognitive functions in the older population. To measure effects of an exergame intervention, a broad variety of outcome measures are reported in the literature (eg, [10]).

Effects on motor or cognitive functions can be assessed by using “traditional” external outcome measures after gameplay such as established paper-pencil tests or questionnaires. A lot of exergames especially commercially available games such as The Nintendo’s Wii Fit provides integrated approaches to evaluate balance performance. Sensors measure bodily movements, and algorithms automatically convert sensor information into quantitative data, for example, the center of pressure (COP) (eg, [10]). The COP is used to control the game tasks, to individually adjusting the gameplay to the user, and also to generate performance scores, which provide the users with instantaneous feedback about game performance in real time (eg, [11]). Scores assessing the users performances have been validated in a few studies but show inconsistent results: One study could show that scores of a step game had good discriminant validity to differentiate between fallers and nonfallers (*P* ≤.001) as well as moderate criterion-related validity (r=−.55 to −.69) and test–retest reliability ranged from poor to good (ICC=.35-.93) in cognitively healthy older adults [12]. In contrast, Wii Fit balance activity scores from different static and dynamic balance tasks performed by “recreationally active” adults (aged 27.0±9.8 years) ranged from poor-to-moderate test–retest reliability (ICC=.29-.74), and concurrent validity was also poor (r<.50) [13]. Goble et al [14] indicate that these results suggest that game software–based assessments displayed as scores are not effective to measure balance ability.

The data flow derived from the game software can also be used to develop specific computerized tests for quantification of performances such as balance control (eg, [15-18]). Although a wide range of results in terms of psychometric properties have been reported as no uniform protocols or outcomes were used for evaluation especially of the Wii Balance Board or the Xbox Kinect [19], validation studies were successful to show that commercially available games basically provide a good basis to create reliable and valid game-based assessments: For example, the Wii Balance Board COP assessment in healthy younger adults showed good-to-excellent test–retest reliability (ICC=.66-.91), inter-rater reliability (ICC=.79-.89), intra-rater reliability (ICC=.70-.92), and concurrent validity (ICC= .73-.89) during single and double limb standing [16,18]. Another study [20] investigated test–retest reliability and construct validity of the Wii Balance Board in people after stroke (mean age 68.3±15.1 years) and showed excellent reliability (ICC=.82-.98) and poor-to-moderate correlations between the Wii Balance Board outcomes and clinical tests. The Wii Balance Board also showed excellent concurrent validity (ICC=.92-.98) with force platform–based assessments during balance tasks in people with Parkinson’s disease [21]. A previous study showed excellent concurrent validity in balance tasks (r>0.75) using the Microsoft Xbox Kinect in healthy adults [17]. Schoene et al [22] have evaluated a custom-made dance mat device to assess stepping reaction times and showed excellent test–retest reliability (ICC=.90) and high correlations with other laboratory assessments (r=.86). Test–retest analyses of assessments using a force platform balance measurement and training device (Good Balance), in which participants had to move their COP along a track (a circle or a zigzag figure) shown on a computer screen, indicated also good results (ICC=.71-.83) [23].

Beyond these commercially available games used for assessment purpose, some researchers have developed and validated own game-based assessment approaches. For example, Szturm et al [24] examined a dual-task computer game–based platform that integrates head tracking and cognitive tasks with balance demands and showed moderate-to-high test–retest reliability (ICC=.55-.75) in healthy, community-dwelling individuals.

### Limitations of Exergame-Assessments Found in the Literature

Although mentioned studies demonstrated that exergames can be used for reliable and valid quantification of motor performances such as balance control, assessment tasks derived from the data stream of a game that show good data on psychometric properties do commonly not represent the original game tasks used for training purpose. Some intervention studies demonstrated that dynamic aspects of COP are typical for game-based balance training requiring the participant to shift their COP to perform tasks such as catching and moving objects or popping rising balloons (eg, [11,15,25,26]). However, to measure changes in balance ability after exergaming, adequate but external instruments (neuropsychological test batteries or functional tests such as the Timed Up and Go [27] or the Berg Balance Scale [28]) have been applied in certain trials (eg, [26,29-31]). Young et al [15] have developed an interface that retrieves information from the Wii Balance Board, which can be used to create a series of balance games for both training and assessment. However, in this study, effects of a training with game tasks developed with the interface (catching apples falling from a tree and popping rising bubbles) were assessed using tasks also created with the interface but which comprised different demands as participants were instructed to maintain a static standing position for 30 seconds with eyes closed and open. Similar internal assessment approaches have also been applied, for example, by Betker et al [25].

Although we could identify 2 studies that have validated assessments based on the game’s original training tasks to obtain a reliable and valid feedback of balance ability during gameplay [22,23], assessment software derived from the data stream of a game commonly includes tasks other than the original game task used for training. Therefore, some of the data might be only loosely associated (eg, use of Timed Up and Go or single or double limb standing with eyes closed to evaluate game-based training gained for shifting the COP while standing) as most validated assessment tasks relate to similar but not identical or integrated performances trained by using exergames.

Despite an increasing number of validation studies evaluating commercially available games or research grade systems not only in young participants without any injuries and history of neurological and musculoskeletal diseases [16-18,24] but also in patients with Parkinson’s disease [21], patients after stroke [20], or frail nursing home residents [23], there is a lack of validation studies including people with diagnosed dementia. However, this patient sample could be a relevant target group for game-based training programs and assessment. For all identified validation studies including older adults, only a cognitive screening was performed allowing a mere classification of cognitive impairment by clinical screening tools, for example, the Mini Mental State Examination (MMSE) (eg, [32]), the Trail Making Test [22] or the Rapid Dementia Screening Test [12]. In some validation studies, mixed samples in terms of the cognitive impairment level might be examined as participants were inadequately screened for cognitive status or screening process was not described in detail (eg, [13,23,24]). We found only 1 validation study that examined feasibility and test–retest reliability of a force platform assessment in people with diagnosis of Alzheimer’s disease [33]. Participants had to move their COP by shifting their weight to 8 numbered targets presented on a screen. Study results showed acceptable test–retest reliability (ICC=.48-.71). Information regarding dementia diagnoses in this study was collected using the MMSE [34] and the Frontal Assessment Battery [35], but only a small sample (n=14) was included in the study.

As outlined previously, there are a number of validation studies mainly focusing on reliability and validity analyses of assessments incorporated into commercially available exergames or of other game-based systems specifically developed for assessment purpose (16-18,20-24). However, we found no study that has conducted not only reliability and validity analyses but also targeting supplementary analyses on sensitivity to change and feasibility to guarantee high methodological quality of the assessment tools. It is striking that previous validation studies did not present especially sensitivity to change data, although this is a paramount objective for validation to evaluate responsiveness in intervention studies. Feasibility analyses based on documentations of completion rates, reasons for missing responses, and mean completion time of assessment process are also generally lacking. Commonly, questionnaires to measure motivation or subjective rating regarding difficulty of the tasks are applied.

### Summary and Aim of the Present Study

In summary, there are a number of studies demonstrating that exergames can be used for reliable and valid quantification of performances such as balance control and provided good data on psychometric properties. However, (1) assessments often include tasks other than the original game task used for training and therefore relate to similar but not identical or integrated performances trained, (2) people with diagnosed dementia have insufficiently been addressed in validation studies, and 3) studies often lack additional validation analyses such as sensitivity to change to document psychometric properties. The purpose of this study was to complement the pool of validated game-based measurements that have already been reported by a number of evaluations. We have developed task-specific assessment parameters based on data directly derived from the data stream during the game task of a training device (Physiomat), which are therefore direct marker of the training tasks. Parameters test a much more complex performance including the interplay (dual task) between challenging motor and cognitive tasks. This approach much better documents the actual game performance compared with another balance performance documentation (eg, during double limb standing with eyes closed) as used in other studies. We aimed to evaluate this internal assessment approach of the training device (Physiomat) in multimorbid, frail elderly with mild-to-moderate dementia. We present data on construct validity, test–retest reliability, sensitivity to change, and feasibility.

## Methods

### Study Design

The validation study was part of a double-blind randomized controlled trial (ISRCTN37232817) to improve motor-cognitive functions in people with mild-to-moderate dementia. To prevent high test burden in the frail and multimorbid sample, validation measures were split. Assessments for validation were conducted before intervention (T1: construct validity and feasibility) and after the intervention period (T2: sensitivity to change) with repeated measures 2-5 days after T2 (retest: test–retest reliability). The trial was performed according to the Helsinki declaration and was approved by the Ethics Committee of the University of Heidelberg.

### Recruitment

Participants were consecutively recruited including geriatric patients, nursing home residents, and community-dwelling persons. Inclusion criteria were: age>65 years, place of residence <15km from the study center, no severe neurological, cardiovascular or psychiatric disorders, or visual deficits, ability to walk 10 m without using a walking aid and written informed consent (obtained by the patient or by a legal representative). Individuals were screened for cognitive impairment using the MMSE [34]. In those with an MMSE of 17-26 indicating cognitive impairment, a comprehensive neuropsychological assessment was performed based on an established neuropsychological test battery (Consortium to Establish a Registry for Alzheimer's Disease—CERAD) [36] and the Number Connection Test (ZVT-G) [37], a modified version of the Trail Making Test (TMT) [38]. Internationally established criteria for cognitive subperformances as assessed in CERAD were used as further inclusion criteria along with amnestic reports for diagnosis of probable dementia. Patients who met predefined criteria for dementia diagnosis based on CERAD results (cognitive subperformances lay under the 10% percentile of the sample corresponding to a z-value of −1.3) were included in the study.

### Measurement and Data Collection

#### Physiomat Assessment

**Figure 1:**
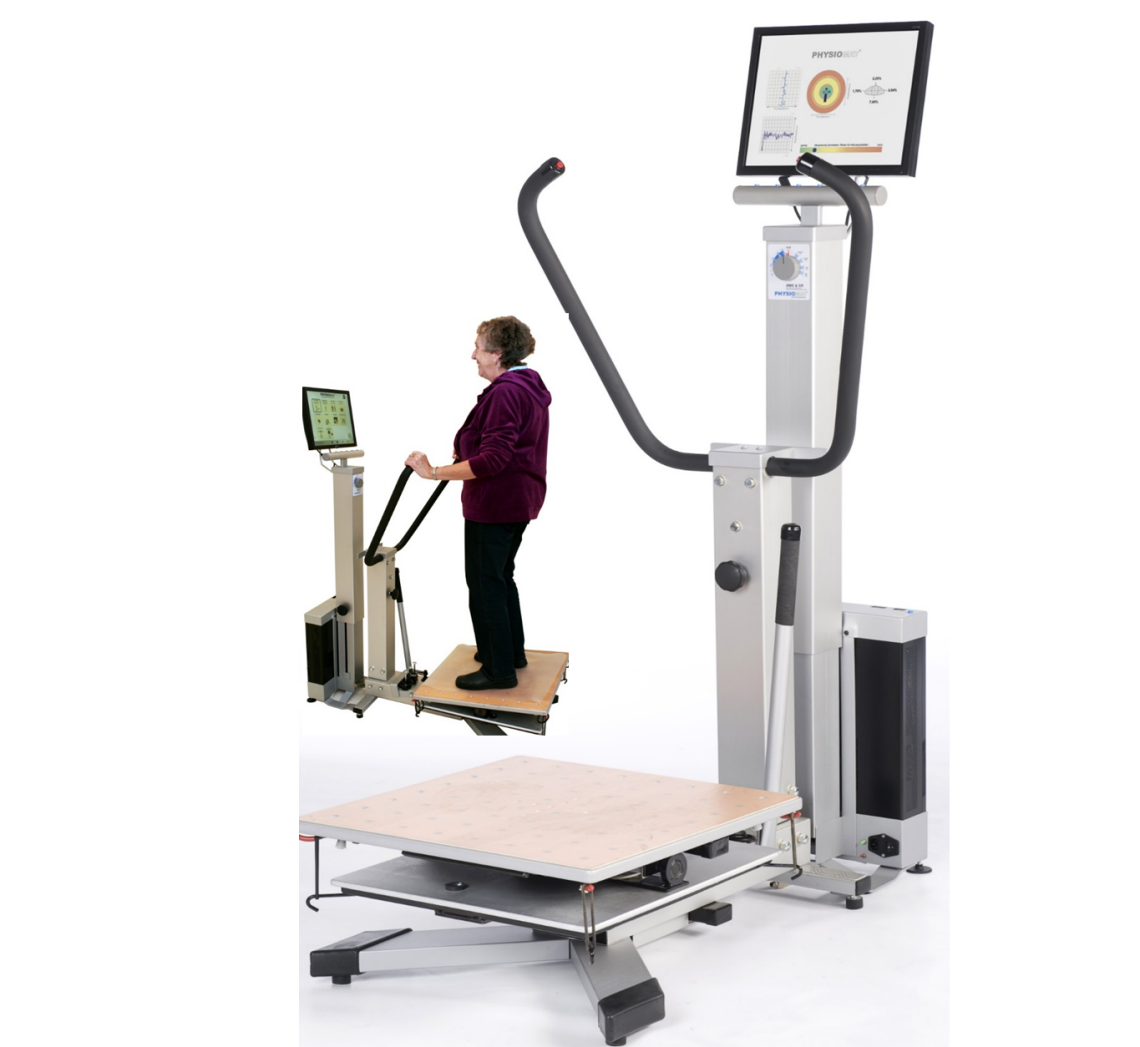
Physiomat including a three-dimensional moveable plate with integrated sensors for displacement measurement. It is connected with a computer and a monitor. Grab rails on each side ensure stability of the patients during training and assessment.

Physiomat (Physio = physiological, M=medical, A=active, T=therapy, EPL medical engineering [39], Figure 1) has been developed as a training device to improve balance performance.

The operating principle of Physiomat based on a specific combination of swivel joints fixed on 2 independent levels enabling bending, tilting, and rotation movements. This device’s construction yields a special 3-dimensional (sagittal, frontal, and transverse level) movement sequences. The internal assessment approach of Physiomat is actually not comparable with the commonly used balance platforms with integrated pressure or inclination sensors. It uses 2 displacement sensors—one for the anterior–posterior motion, the other for the medio–lateral tilting and rotation motion—to record the movements of the platform. This is done by measuring the changes in resistance (measurement range 0-100 kohm, measurement accuracy 0.1 kohm, sampling rate 100 Hz). This sensor information is converted into a standard signal (normed electrical signals) by an analog-digital converter. Standard signal output acquired via the displacement sensors generates digital numerical values (digits) in a measuring range of 0-1000 digits for each motion axis. This means that the movement excursion of the platform is measured in digits/ms, and based on that sway path and sway area are determined as quantitative parameters. Movement excursion of the platform measured in digits/ms is also presented to the participants in terms of a visual feedback on the monitor in real time to control the cursor by mapping it to the target motion to solve Physiomat tasks.

The software with the training and assessment tasks as used in this study was specifically developed by the research group of the AGAPLESION Bethanien Hospital Heidelberg in cooperation with EPL to target motor-cognitive performances in patients with dementia. The assessment strategy derived from the data stream of Physiomat game tasks. The Physiomat assessment linked cognitive and motor-functional demands; concurrent dual tasks of various elements on balance ability (weight-shifting tasks and postural control while standing) with specific cognitive subperformances such as executive functions are required.

To provide a motor-cognitive task to test a complex performance including the interplay (dual task) between challenging balance and cognitive tasks, the Physiomat-Trail-Making Tasks (PTMTs) have been developed. Compared with other exergames (eg, Nintendo Wii) including virtual reality tasks where an avatar is displayed on the screen that follows the participant’s movements that do commonly not coincide with evidence-based neuropsychological tasks, we incorporated an internationally established cognitive test (the Trail-Making Test) into a balance training device. This test has been modified and successfully validated for use in older and cognitively impaired persons [37] with the introduction of a learning phase using an increasing number of digits before testing and reducing the complexity of the task by positioning of the digits. This modified version prevents frequent floor effects as compared by the original tests and is valid for the target sample of this study of cognitively impaired persons. The test is sensitive also for early deficits in the course of neurodegenerative diseases and documents cognitive subperformances such as executive functions including procedural memory, visual–spatial orientation, and attention-related performances (especially in the test setting as used in this study with the concurrent dual task of balance control with the specific cognitive subperformance of divided attention, see in the following section). These cognitive subperformances appear relatively early in the course of the disease and are therefore an adequate test for the study sample of patients with mild-to-moderate stage dementia.

**Figure 2:**
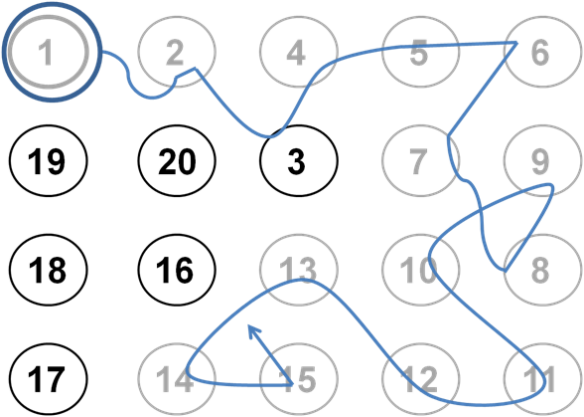
Example for complex Physiomat Trail-Making Task (PTMT). Participants were instructed to capture digits in correct order as fast as possible by shifting the weight to numbered targets.

PTMTs include different performance levels as defined by the number of digits to be tested. The participants were instructed to move the cursor on the screen (indicating the participant’s bending, tilting, and rotation motion) directly to each numbered target with the aim to connect an increasing number of digits (number of digits: 4, 7, 9, 14, 20) as fast as possible by weight shifting (Figure 2). Physiomat platform was not fixed and allows movements in all directions, which must be controlled by the user. The degree of movements is partly limited by rubber rings attached to the corners of the platform. We used several rings to achieve a feasible motor challenge but did not modify this for the rather homogeneous sample with impaired motor status, advanced age, and multimorbidity including cognitive impairment. Participants were instructed to use handles (see Figure 1) to control movements. For validation purpose, we only used results of the simple (4 digits), moderate (9 digits), and complex (20 digits) PTMT, as we assumed that this range of complexity levels would be sufficient for the study purpose. With the standardized motor task and the standardized but increasing challenge level of the cognitive task, we ascertained a standardized assessment procedure.

**Figure 3:**
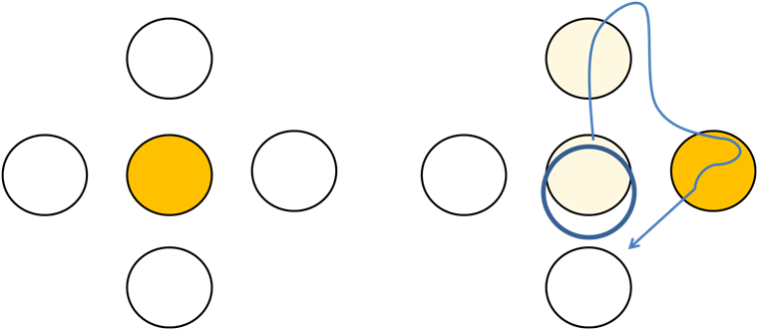
Physiomat Follow-the-Ball Task (FTBT). Participants were instructed to follow a yellow ball during displacement of center of mass as fast as possible using the handles.

We also applied an additional standardized motor task without an increasing challenge level of cognitive task to study psychometric properties. Instructions were to move the cursor from the center of the screen directly to the targets highlighted as a moving yellow ball on the screen as fast as possible. This Follow-The-Ball Task (FTBT) was used to assess participants’ ability to move their center of mass by shifting their weight to the highlighted targets (Figure 3).

**Figure 4:**
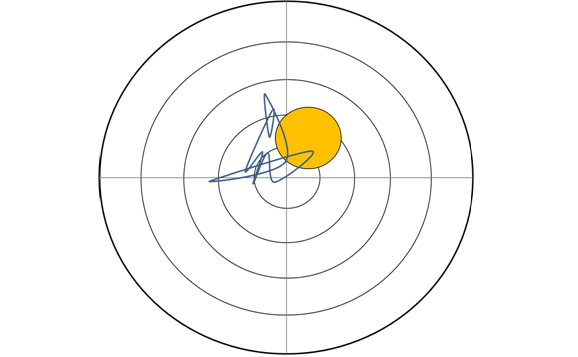
The Physiomat Balance Task (PBT). Participants were instructed to stand still on the plate and keep in the middle of a yellow target for 3, 10 and 30 seconds without using the grab rails.

We also used 3 Physiomat balance tasks (PBTs) challenging postural control while standing without using the handles (Figure 4). Tasks also differed in complexity levels (keeping postural control for 3, 10, and 30 seconds). The platform was also not fixed but contrary to the PTMTs and the FTBTs, the degree of movements was limited by a larger number of rubber rings.

During assessment, no physical assistance or cueing was allowed. For each Physiomat measurement, the best performance of 2 trials was used for statistical analyses. Temporal (test duration in seconds) and spatial (sway path in mm/s, sway area in mm^2^/s) parameters have been documented as main study end points of assessment.

Furthermore, we documented the number of successfully performed Physiomat tasks for each measurement by doing dichotomous coding (1=successful; 0=not successful). Based on that we calculated a scoring for PTMT (PTMT score), for PBT (PBT score), and for the complete Physiomat assessment (total score) by summarizing the numerical codings. For PTMT score and PBT score, up to 3 points could be achieved for each as there were 3 levels (PTMTs: 4, 9, and 20 digits; PBTs: 3, 10, and 30 seconds) indicating that all complexity levels have been performed successfully. For the total score including all PTMTs, PBTs, and the FTBT, a maximum of 7 points could be achieved.

#### Descriptive Measures

Demographic and clinical characteristics including age, gender, education, social status (independent or institutionalized), number of falls in the previous year, and number of medications and diagnoses were documented. Psychological status was assessed by the Geriatric Depression Scale [40] for depressive symptoms and by the Falls Efficacy Scale-International (FES-I) [41] for fall-related self-efficacy. Motor-functional status was assessed by the Timed-Up and Go (TUG) [27] and performance-oriented mobility assessment (POMA) [42]. Cognitive status was screened by the MMSE [34].

The TUG, POMA, and MMSE were also used for validity analyses. The following tests were additionally used for testing construct validity: The simple Physiomat balance task (10 seconds) and the FTBT as Physiomat balance tests (Figures 3 and 4) and word list immediate recall [43] as a subtest of the CERAD, the ZVT-G, and repeating numbers (ZN-G) [37].

### Statistical Analysis

Statistical analyses were performed on SPSS 22.0 for Windows. Descriptive data are presented as means and standard deviations (SD) or number and percentages (%) as appropriate. The Kolmogorov–Smirnov test and histograms were used to analyze distribution of data. In case data were not normally distributed, nonparametric tests were used in addition to parametric tests.

#### Construct Validity

Spearman’s rank correlations between temporal parameters (test duration in seconds) of simple, moderate, and complex PTMTs and theoretically related motor-functions, respectively, cognitive measures as described previously were calculated to test 8 predefined hypotheses [44,45]. The hypotheses are given in detail in Table 3. Our assumptions can be summarized briefly as follows:

Cognitive measures as MMSE and especially ZVT-G were expected to be associated with PTMTs because both motor-cognitive Physiomat tasks and the mentioned tests require multiple cognitive abilities. A higher correlation with ZVT-G was expected because both tests assess a similar construct where attentional abilities and executive functions are demanded. In comparison, cognitive instruments measuring domain-related cognitive functions such as memory abilities (immediate wordlist recall and number repeating) were expected to be less associated with PTMTs.

Established motor-functional assessments (TUG and POMA) measuring postural control and gait performance were expected to be associated with motor-cognitive Physiomat tasks as also balance performances are demanded in each of the tests. The FTBT was expected to be highly correlated with all PTMTs because both tasks are performed during weight shifting.

Regarding the complexity levels of motor-cognitive Physiomat tasks, we expected that complex PTMTs were strongly associated with cognitive measures and simple PTMTs with motor-functional outcomes because of an increasing cognitive challenge by accelerating complexity level.

#### Test–Retest Reliability

Test–retest assessments were performed within 2-5 days by the same examiner to exclude interobserver variability. For test–retest analyses, Spearman’s rank correlations were calculated according to Cohen’s criteria [46] for low (r_s_ < .2), moderate (r_s_ = .2-.5), or high (r_s_ > .5) correlations. In addition, we used ICC coefficients using a 2-way mixed model [47]. For ICCs, 95% confidence intervals were given. ICCs were considered as low (ICC <.40), moderate (.40 ≤ ICC ≤.75), or high (ICC >.75) [48].

#### Sensitivity to Change

To study the responsiveness of trained Physiomat tasks, we used baseline values of a RCT (ISRCTN37232817), which will be published in the near future. Progressive Physiomat training (10 minutes twice a week) in 47 participants was part of this comprehensive intervention (1.5 hours, twice a week for 10 weeks) in groups at the maximum of 7 participants including dual tasking (walking while counting) and training of compensatory sit to stand movement maneuvers to improve motor-cognitive abilities in people with dementia. Subjects of the control group (n=43) underwent a supervised, unspecific motor-functional group training for 10 weeks (1 hour, twice a week) including low-intensity strength training and flexibility exercises for the upper limbs while seated. In this paper, only the results of the intervention group that conducted the Physiomat training as one part of the overall training program was used to document sensitivity to change.

Physiomat exercise sessions were composed of the FTBT introducing the participants to Physiomat and to provide relevant strategies of balance displacement. In addition, PTMTs were trained, and complexity level was gradually increased according to the capacity of each participant. Physiomat balance tasks were not part of the intervention and responsiveness analyses. The Wilcoxon test was applied to test sensitivity to change. Effect sizes were calculated using standardized response means (SRMs) [44] according to Cohen’s criteria (small effect < .2, moderate effect .2 ≤ SRM > .5, and large effect .8 and above) [46].

#### Feasibility

To study feasibility of motor-cognitive (PTMTs) and motor-functional (PBT and FTBT) Physiomat measures, percentages of successfully completed tasks (completion rates), reasons of missing responses, and the mean completion time as measured by a stopwatch were documented. To assess safety of the participants, issues such as a slip or fall and any clinical events during testing were systematically documented.

It should be noted, that only 7 of 15 Physiomat tasks assessed for further analyses were used for validation purpose. Documentation of feasibility outcomes was related to the overall Physiomat assessment protocol, which additionally included a comprehensive instruction period and breaks between single performance levels for the frail, multimorbid, and cognitively impaired participants. Therefore, completion time with regard to the assessments to test feasibility will be overestimated.

## Results

### Participants’ Characteristics

The study sample included 105 (mean age 82.7±5.9) multimorbid and cognitively impaired subjects living at home or in nursing homes. Further demographic and clinical characteristics are summarized in Table 1.

**Table 1:** Descriptive characteristics of the participants.^a^

Characteristics^b^	All participants N=105
Age (years), mean (SD)	82.7 (5.9)
Gender (female), n (%)	76 (72.4)
Education (years), mean (SD)	11.8 (2.9)
Social status (institutionalized), n (%)	31 (29.5)
Cognitive status MMSE^b^ (sum score), mean (SD)	21.9 (2.8)
Depression GDS^c^ (sum score), mean (SD)	2.8 (2.3)
Indicated depression (GDS score >5), n (%)	19 (18.1)
Number of falls, n (%)	49 (46.7)
Fear of falling FES-I^d^ (sum score), mean (SD)	9.2 (2.8)
Number of diagnosis, mean (SD)	8.2 (4.1)
TUG^e^ (test duration in seconds)	18.4 (11.3)
POMA^f^ (total score)	22.3 (4.0)

^a^ Given are sample size (N), mean and standard deviation (SD) or percentages (%) of the sample of all characteristics.

^b^ MMSE: Mini-Mental-State Examination

^c^ GDS: Geriatric Depression Scale

^d^ FES-I: Falls Efficacy Scale International

^e^ TUG: Timed Up and Go

^f^ POMA: Performance-Oriented Mobility Assessment

We separated the validation measures into 3 assessment sessions (feasibility and construct validity analyses at baseline, sensitivity to change measures after the intervention, and test–retest reliability assessment within the subsequent 2-5 days) to prevent high test burden. Assessments for feasibility and construct validity analyses were not practicable for 6 of 105 subjects (5.7%) because of serious motor-functional disability (n=3), visual impairment (n=2), and fear of assessment using Physiomat (n=1). Sensitivity to change was assessed in 47 participants in a subsample of the intervention group (n=56) as 9 participants (16.1%) dropped out owing to physical limitations (n=3), noncompliance (n=5), and death (n=1). Test–retest reliability could not be assessed in 31 of 105 participants (29.5%) because of physical limitations and pain (n=11), noncompliance (n=13), death (n=4), and increased visual impairment (n=3).

### Construct Validity

PTMTs have shown a high association with established cognitive paper-and-pencil tests (ZVT-G and MMSE) and moderate associations with motor-functional instruments (TUG and POMA) indicating a good construct validity of motor-cognitive Physiomat outcomes. Correlations between simple, moderate, and complex PTMTs and cognitive as well as motor-functional paper-and-pencil tests are illustrated in Table 2.

**Table 2:** Construct validity of motor-cognitive Physiomat tasks.^a^

Test	Variable (unit)	Simple PTMT^b^ (*P* value)	Moderate PTMT (*P* value)	Complex PTMT (*P* value)
FTBT^c^	Duration (time in seconds)	68^d^ (*P* ≤.001)	.71^d^ (*P* ≤.001)	.61^d^ (*P* ≤.001)
PBT^e^ 10 sec.	sway path (mm/second)	0.11^f^ (*P*=.31)	−0.03^f^ (*P*=.79)	−0.34^g^ (*P*=.10)
POMA^h^	Total score	−0.22^g^ (*P*=.03)	−0.40^g^ (*P* ≤.001)	0.08^f^ (*P*=.71)
TUG^i^	Duration (time in seconds)	0.22^g^ (*P*=.03)	0.48^g^ (*P* ≤.001)	0.19^f^ (*P*=.35)
MMSE^j^	Total score	0.29^g^ (*P*=.004)	0.35^g^ (*P*=.002)	0.66^d^ (*P* ≤.001)
ZVT-G^k^	Duration (time in s)	0.36^g^ (*P* ≤.001)	0.44^g^ (*P* ≤.001)	0.83^d^ (*P* ≤.001)
ZN-G^l^	Total score	−0.25^g^ (*P*=.02)	−0.19^f^ (*P*=.12)	−0.22^g^ (*P*=.30)
Wordlist immediate recall	Number of quoted words	−0.33^g^ (*P*=.004)	−0.42^g^ (*P*=.04)	−0.16^f^ (*P*=.12)

^a^ Given are Spearman’s rank correlations (r_s_) between simple (4 numbers), moderate (9 numbers), and complex (20 numbers) PTMTs and motor-functional (FTBT, moderate PBT, POMA, and TUG) and cognitive outcomes (MMSE, ZVT-G, ZN-G and wordlist immediate recall).

^b^ Physiomat Trail-Making Task

^c^ FTBT: Follow-The-Ball Task

^d^ High correlation (r_s_ > .5)

^e^ PBT: Physiomat-Balance-Task

^f^ Low correlation (r_s_ < .2)

^g^ Moderate correlation (.2 ≥ r_s_ ≤ .5)

^h^ POMA: performance-oriented mobility assessment

^i^ TUG: Timed Up and Go

^j^ MMSE: Mini-Mental-State Examination

^k^ ZVT-G: modified version of the Trail-Making-Test A

^l^ ZN-G: repeating numbers

Correlations between measures assessing motor-functional performances (TUG, POMA, FTBT, moderate PBT—10 seconds) and PTMTs were low to high (r_s_= −.03-.71). Highest correlations (*P* ≤ .001) were found for duration of FTBT (r_s_=.61-.71). Correlations with PBT, POMA, and TUG were low to moderate (r_s_= −.03 to .48). Highest correlations with cognitive outcomes (*P* ≤ .001) were found between complex PTMT and MMSE (r_s_=.66) and ZVT-G (r_s_=.83). Correlations with instruments measuring memory skills were low to moderate (ZN-G: r_s_= −.19 to −.25; wordlist immediate recall: r_s_= −.16 to −.42).

Construct validity assessed by testing 8 a priori hypotheses is presented in Table 3. Except hypothesis number 8, all assumptions could be confirmed (87.5%) for PTMTs regarding temporal parameters (time in seconds) indicating excellent construct validity [45].

**Table 3:** Results of 8 predefined hypotheses.

No.	Hypothesis^a^ Expected associations with cognitive outcome measures	Hypothesis confirmed?
1	We expected moderate-to-high associations between PTMTs^b^ and MMSE^c^ as both assessments measure multiple cognitive functions.	Yes
2	We expected more pronounced associations between PTMTs and ZVT-G^d^ as both assessments measure a similar construct where particularly attentional abilities are demanded.	Yes
3	We expected moderate associations between PTMTs and memory tests as both cognitive tests would cover different cognitive subperformances as compared with PTMTs.	Yes
4	We expected higher associations of cognitive outcome measures with increasing complexity of PTMTs as for difficult Physiomat levels cognitive demands may predominate.	Yes (except ZN-G^e^)
	Expected associations with motor-functional outcome measures	
5	We expected associations between PTMTs and TUG^f^ as well as POMA^g^ as also balance performances are demanded in each of the assessments, although not comparable in type of assessment.	Yes
6	We expected pronounced associations between PTMTs and FTBT^h^ as FTBT is a preliminary Physiomat training task requiring similar strategies of balance performances.	Yes
7	We expected a less association between PTMTs and the moderate PBT^i^ (10 seconds) as this Physiomat task requires a different strategy of balance performance.	Yes
8	We expected higher associations of motor-functional outcomes with decreasing complexity of PTMTs as for simple Physiomat levels motor-functions demands may predominate.	No

^a^ Hypotheses are given for Spearman’s rank correlations between PTMTs, motor-functional outcomes (hypotheses 5-8), and cognitive outcomes (hypotheses 1-4) of selected comparison measurement instruments.

^b^ PTMT: Physiomat Trail-Making Task

^c^ MMSE: Mini-Mental-State Examination

^d^ ZVT-G: modified version of the Trail-Making-Test A

^e^ ZN-G: repeating numbers

^f^ TUG: Timed Up and Go

^g^ POMA: performance-oriented mobility assessment

^h^ FTBT: Follow-The-Ball Task

^i^ PBT: Physiomat-Balance Task

### Test–Retest Reliability

For almost all outcomes of Physiomat measures and for requirement level concerning all Physiomat tasks (total, PBT, and PTMT score) moderate-to-high correlations between test and retest assessment were found indicating good to excellent test–retest reliability (Tables 4 and 5).

**Table 4:** Test–retest results of all Physiomat tasks and requirement level (Spearman’s rank correlations).

Test	Variable (unit)	N	Mean (SD) Test	Mean (SD) Retest	r_s_	*P* value
PBT^a^ 3 Sec						
	Sway path (mm/second)	71	134.4 (83.9)	120.6 (84.1)	.48^b^	≤.001
	Sway area (mm^2^/second)		627.7(1311.3)	534.7(1289.1)	.45^b^	≤.001
PBT 10 Sec						
	Sway path (mm/second)	68	571.3 (312.2)	568.7 (292.6)	.68^c^	≤.001
	Sway area (mm^2^/second)		654.5 (1164.8)	627.7 (1120.2)	.60^c^	≤.001
PBT 30 Sec						
	Sway path (mm/second)	61	1719.3 (1020.5)	1589.8 (844.1)	.78^c^	≤.001
	Sway area (mm^2^/second)		750.2 (1729.2)	563.7 (1015.2)	.75^c^	≤.001
FTBT^d^						
	Sway path (mm/second)	73	3449.1 (1044.2)	3269.1 (1005.5)	.74^c^	≤.001
	Duration (time in seconds)		20.9 (5.4)	21.2 (6.8)	.69^c^	≤.001
Simple PTMT^e^	Sway path (mm/second)	73	1883.7 (558.5)	1774.7 (343.4)	.59^c^	≤.001
	Duration (time in seconds)		8.2 (2.8)	8.2 (2.9)	.60^c^	≤.001
Moderate PTMT						
	Sway path (mm/second)	69	3722.3 (910.9)	3630.9 (923.8)	.78^c^	≤.001
	Duration (time in seconds)		20.8 (5.9)	19.9 (6.2)	.74^c^	≤.001
Complex PTMT			
	Sway path (mm/second)	47	8319.4 (2220.8)	8111.1 (2170.9)	.80^c^	≤.001
	Duration (time in seconds)		51.0 (16.2)	49.1 (16.7)	.87^c^	≤.001
Total Score		74	6.3 (1.1)	6.3 (1.1)	.89^c^	≤.001
PTMT Score		74	2.6 (0.6)	2.6 (0.6)	.89^c^	≤.001
PBT Score		74	2.8 (0.7)	2.7 (0.7)	.87^c^	≤.001

^a^ PBT: Physiomat-Balance Tasks

^b^ moderate correlation (.2 ≥ r_s_ ≤ .5)

^c^ high correlation (r_s_ > .5)

^d^ FTBT: Follow-The-Ball Task

^e^ PTMT: Physiomat Trail-Making Task

Regarding Spearman’s rank correlations reliability for the total sample was moderate to high (r_s_=.45-.89) for all variables. Highest correlations were found for sway path (r_s_=.80) and duration (r_s_=.86) of the complex PTMT as well as for requirement level (total score r_s_=.89; PTMT score r_s_ =.89; and PBT score r_s_=.87). Moderate correlations were only found for sway path (r_s_ =.48) and sway area (r_s_=.45) of the simple PBT (3 seconds). Overall, it could be observed that correlations increased with the duration of PBT and the complexity of PTMT.

**Table 5:** Test–retest results of all Physiomat tasks and requirement level (ICCs^a^).

Test	Variable (unit)		N		Mean (SD) Test		Mean (SD) Retest			ICC (95%CI)		*P* value
S												
	Sway path (mm/second)		71		134.4 (83.9)		120.6 (84.1)			.50^b^ (.30-.66)		≤.001
	Sway area (mm^2^/seconds)				627.7 (1311.3)		534.7 (1289.1)			.73^b^ (.59-.82)		≤.001
PBT^c^ 10 Sec												
	Sway path (mm/second)		68		571.3 (312.2)		568.7 (292.6)			.66^b^ (.50-.78)		≤.001
	Sway area (mm^2^/s)				654.5 (1164.8)		627.7 (1120.2)			.57^b^ (.38-.71)		≤.001
PBT 30 Sec						
	Sway path (mm/seconds)		61		1719.3 (1020.5)		1589.8 (844.1)			.73^b^ (.59-.83)		≤.001
	Sway area (mm^2^/seconds)				750.2 (1729.2)		563.7 (1015.2)			.32^d^ (.08-.53)		.005
FTBT^e^												
	Sway path (mm/second)		73		3449.1 (1044.2)		3269.1 (1005.5)			.84^f^ (.76-.89)		≤.001
	Duration (time in seconds)				20.9 (5.4)		21.2 (6.8)			.79^f^ (.68-.86)		≤.001
Simple PTMT^g^												
	Sway path (mm/second)		73		1883.7 (558.5)		1774.7 (343.4)			.47^b^ (.27-.63)		≤.001
	Duration (time in seconds)				8.2 (2.8)		8.2 (2.9)			.55^b^ (.37-.69)		≤.001
Moderate PTMT												
	Sway path (mm/second)		69		3722.3 (910.9)		3630.9 (923.8)			.74^b^ (.61-.82)		≤.001
	Duration (time in seconds)				20.8 (5.9)		19.9 (6.2)			.79^f^ (.68-.87)		≤.001
Complex PTMT												
	Sway path (mm/second)		47		8319.4 (2220.8)		8111.1 (2170.9)			.82^f^ (.69-.89)		≤.001
	Duration (time in seconds)					51.0 (16.2)		49.1 (16.7)		.83^f^ (.72-.91)		≤.001
Total score												
	Score		74		6.3 (1.1)		6.3 (1.1)			.92^f^ (.88-.95)		≤.001
PTMT score												
	Score		74		2.6 (0.6)		2.6 (0.6)			.90^f^ (.85-.94)		≤.001
PBT score												
	Score		74		2.8 (0.7)		2.7 (0.7)			.89^f^ (.84-.93)		≤.001

^a^ ICC: intraclass correlations

^b^ moderate ICC (.40 ≤ ICC ≤.75)

^c^ PBT: Physiomat-Balance Tasks

^d^ Low ICC (< .40)

^e^ FTBT: Follow-The-Ball Task

^f^ high ICC (ICC >.75)

^g^ PTMT: Physiomat Trail-Making Task

Regarding ICCs, moderate-to-high test–retest reliability (ICC=.47-.92) was found for almost all variables. Only sway path of the complex Physiomat balance task (30 seconds) was below the threshold of moderate reliability (ICC <.40). High ICCs were found for sway path (ICC=.82) and duration (ICC=.84) of not only the complex PTMT and for requirement level (total score ICC=.92; PTMT score ICC=.90; PBT score ICC=.89) but also for the duration of the moderate PTMT (ICC=.79). ICCs increase with complexity level of PTMT. For sway path (ICC=.84) and duration (ICC=.79) of FTBT, high correlations were proven, too.

Spearman’s correlations and ICCs were all significantly different from zero at a.01 level (*P* ≤ .001).

To examine potential influence of deviating subsamples in different conditions (larger sample in simple condition, selection to high functioning participants in more complex conditions) on test–retest reliability, we conducted a subsequent test–retest analysis of 47 participants, which successfully conducted all complexity levels (Table 6).

**Table 6:** Subanalysis of test–retest reliability of motor-cognitive Physiomat tasks.

Test	Variable (unit)	N^a^	Mean (SD) Test	Mean (SD) Retest	ICC (95%CI)	*P* value
Simple PTMT^b^						
	Sway path (mm/second)	47	1799.9 (417.6)	1793.1 (313.9)	.36^c^ (.09-.59)	.006
	Duration (time in seconds)		7.3 (2.2)	7.5 (2.5)	.44^d^ (.18-.64)	.001
Moderate PTMT						
	Sway path (mm/second)	47	3660.4 (675.4)	3602.8 (674.5)	.75^d^ (.59-.85)	≤.001
	Duration (time in seconds)		19.2 (4.2)	18.6 (4.3)	.79^e^ (.66-.88)	≤.001
Complex PTMT						
	Sway path (mm/second)	47	8392.7 (2248.3)	8204.9 (2145.3)	.81^e^ (.69-.89)	≤.001
	Duration (time in seconds)		51.0 (16.2)	49.1 (16.7)	.84^e^ (.72-.91)	≤.001

^a^ Subanalysis of test–retest reliability was conducted in a subsample of 47 participants, which successfully conducted all complexity levels of PTMTs.

^b^ PTMT: Physiomat Trail-Making Task

^c^ Low ICC (< .40)

^d^ Moderate ICC (.40 ≤ ICC ≤.75)

^e^ High ICCs (ICC >.75)

Results are comparable to the results of the nonselected group. Moderate-to-high test–retest reliability (ICC=.44-.84) was found for almost all variables. Only sway path of the simple PTMT was below the threshold of moderate reliability (ICC<.40). ICCs also increase with complexity level of PTMT.

### Sensitivity to Change

All trained Physiomat tasks (FTBT and PTMTs) showed significant improvements indicating good-to-excellent sensitivity to change. Results of the Wilcoxon test and effects sizes (SRMs) are outlined in Table 7.

**Table 7:** Sensitivity to change for trained Physiomat tasks.

Test	Variable (unit)	N	Mean (SD) T1 – before intervention period	Mean (SD) T2 – after intervention period	*P* value^a^	SRM^b^
FTBT^c^						
	Sway path (mm/second)	47	4356.5 (3064.8)	3135.4 (569.6)	≤.001	0.4^d^
	Duration (time in seconds)		19.3 (4.6)	18.6 (4.3)	≤.001	0.7^e^
Simple PTMT^f^ (4 numbers)						
	Sway path (mm/second)	45	2944.3 (4597.5)	1732.5 (307.3)	≤.001	0.3^d^
	Duration (time in seconds)		17.6 (21.9)	7.2 (1.9)	≤.001	0.5^e^
Moderate PTMT (9 numbers)						
	Sway path (mm/second)	37	4296.5 (1482.6)	3472.5 (643.1)	≤.001	0.7^e^
	Duration (time in seconds)		28.6(10.6)	18.5(4.1)	≤.001	1.1^g^
Complex PTMT (20 numbers)						
	Sway path (mm/second)	14	8361.7 (2269.5)	6850.6 (1341.2)	.01	0.8^e^
	Duration (time in seconds)		57.6 (11.7)	37.5 (7.8)	.001	2.0^g^
PTMT Score		47	2.0 (0.8)	2.8 (0.6)	≤.001	1.1^g^

^a^*P* values for Wilcoxon test applied to test differences between T1 and T2.

^b^ SRM: standardized response mean (difference between the mean scores at assessments, divided by the mean scores of the standard deviation).

^c^ FTBT: Follow-The-Ball Task

^d^ Small effect size (SRM=0.2-0.5)

^e^ Moderate effect size (SRM=0.5-0.8)

^f^ PTMT: Physiomat Trail-Making Task

^g^ Large effect size (SRM >0.8)

Results showed significant changes (*P* ≤ .001) of both spatial and temporal parameters of PTMTs and the FTBT after a 10-week Physiomat intervention (twice a week/10 minutes). Large effect sizes were evident especially for duration (SRM=2.0) of complex PTMT and moderate PTMT (SRM=1.1). There were also significant changes (*P* ≤ .001) for requirement level concerning motor-cognitive Physiomat tasks (PTMT score) with a large effect size (SRM=1.1).

### Feasibility

Physiomat assessment was found to be practicable by frail, old, and multimorbid persons with mild to moderate–stage dementia. There were no clinical events, slips, or falls during training or assessment. A total of 99 of 105 participants (94.3%) could be tested at baseline. Five subjects could not be assessed due to severe motor-functional (hemiparesis) and visual (blindness) limitations. Willingness of the participants to attend to the computerized Physiomat assessment was excellent, and only 1 very frail and fearful person refused assessment.

**Figure 5:**
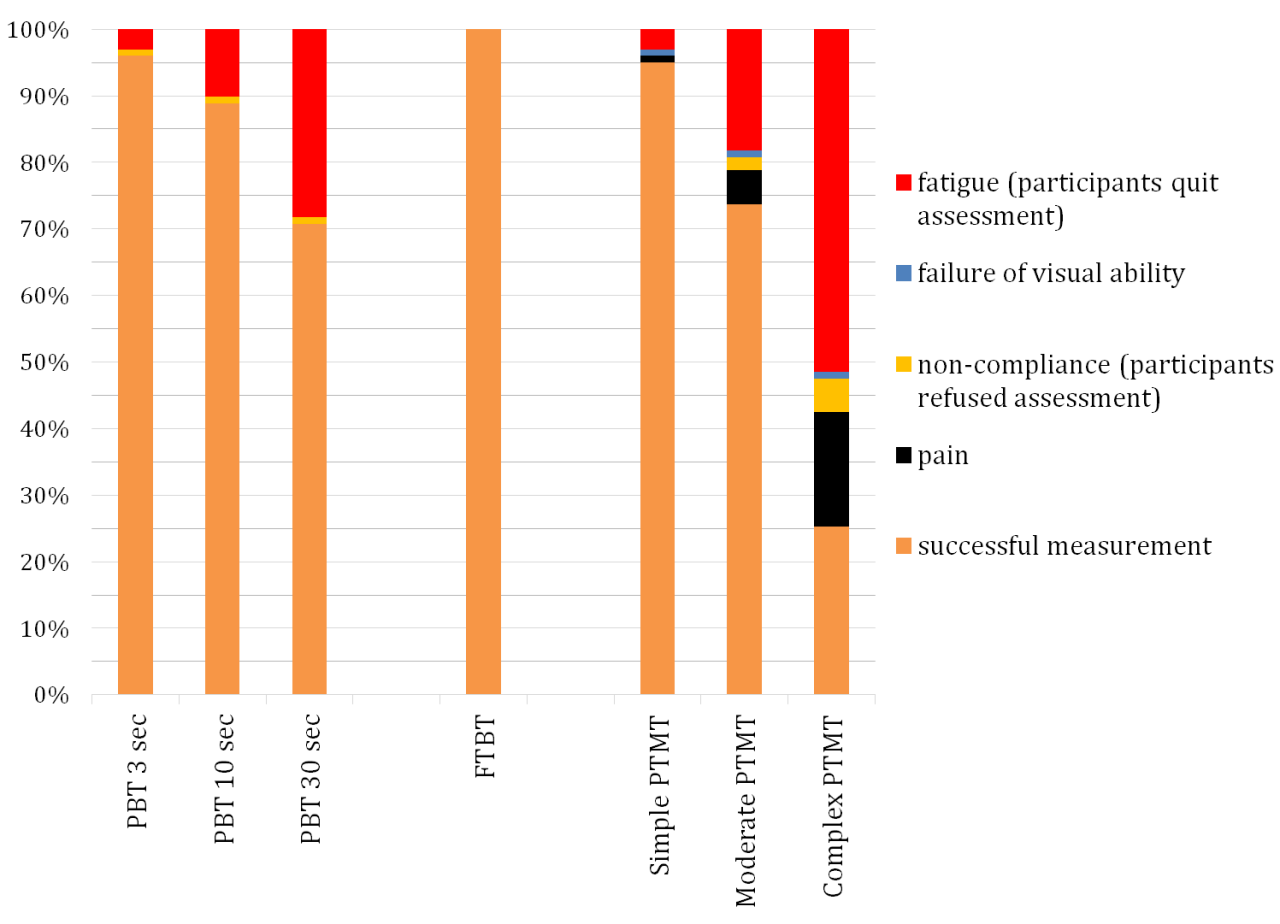
Feasibility analysis including response rates during a consecutive Physiomat assessment.

Duration of assessment averaged 25.8 minutes (range: 6-55 minutes). No technical problems in assessing and analyzing the data occurred. Results of response rates are illustrated in Figure 5. For most of the participants, PBT (3, 10, and 30 seconds) was feasible. 30 of 99 subjects (30.3%) could not execute the complex PBT (30 seconds) because of self-reported fatigue. All participants could perform the FTBT. Regarding the motor-cognitive Physiomat tasks, 94 of 99 participants (94.9%) could conduct the simple, 73 subjects (73.7%) the moderate and 25 subjects (25.3%) the complex PTMT. Reasons for discontinuation were also predominantly fatigue reported by almost half of the participants (51.5%) followed by pain (17.2%) and by noncompliance (5.1%).

## Discussion

### Principal Findings

In this study, we validated an internal assessment approach of a game-based training device (Physiomat) to obtain a reliable and valid feedback of motor-cognitive abilities during gameplay. In contrast to recent studies focusing on computerized game-based assessments, which often conducted only reliability and validity analyses, we investigated multiple psychometric properties to allow a more comprehensive evaluation of the methodological quality of the assessment tested. In this study, validation was performed in frail, older persons with mild-to-moderate dementia who had not been addressed in most previous validation studies. Despite the crucial problems to assess persons with dementia [49], results indicated good construct validity, test–retest reliability, sensitivity to change, and feasibility of the tested device.

#### Construct Validity

In this study, construct validity was analyzed by testing 8 a priori hypotheses. For this purpose, Spearman‘s rank correlations between temporal parameters (test duration in seconds) of simple, moderate, and complex PTMTs and theoretically related motor-functional as well as cognitive measures were calculated. Almost all predefined hypotheses could be confirmed indicating a good construct validity of Physiomat assessment.

Expected higher correlations of cognitive instruments with increasing complexity of PTMTs were found for all cognitive outcome measures except the ZN-G. Correlations with ZN-G and wordlist immediate recall were partly not significant. As highest correlations were found between the complex PTMT and ZVT-G, more difficult motor-cognitive tasks seem to be associated with increased cognitive demands especially including attentional abilities and information processing. These results showed that particularly domain-related cognitive functions could rather be assessed when participants perform more complex PTMTs.

To our knowledge, there are no studies including cognitively impaired participants that investigated the relationship between interactive computerized assessment strategies comparable to Physiomat and cognitive test batteries, which do not use technological devices, although game-based assessments require a combination of motor and cognitive abilities. Expected higher associations of motor-functional instruments with decreasing complexity of PTMTs could not have conclusively identified for all tests (Table 2). Except the Physiomat balance task, highest correlations of motor-functional tests (FTBT, TUG, and POMA) were actually found with moderate and simple PTMTs, and expected lowest associations between complex PTMTs and TUG as well as POMA were not significant. However, when balance performances are challenged, rather an additional complex cognitive task than a simple or moderate task may require a higher level of attention in patients with dementia, which leads to a decrease in postural control. A previous study [50] found a further decline of up to 15% in postural control during a more complex task in a cognitively impaired sample. Although findings were not significant, which might be due to a small study sample, balance performances seem to be determined by the complexity level of additional cognitive tasks, which could explain stronger correlations between motor-functional outcome measures and easier levels of PTMTs in our study.

We could not find any studies that have investigated the relationship between interactive assessment methods and established motor tests in people with dementia. Therefore, the comparability of our results with recent validity studies is limited. One study [20] examined associations of the Wii Balance Board and clinical tests in patients after stroke. The study could show moderate Spearman’s correlations (r_s_=−0.57) between a Wii Balance Board dynamic balance task measuring shifting of body weight to follow a visual feedback target and the TUG, which is comparable to our findings. A further study examining the association of TUG scores and different levels of a computer-based balance board test using the Biodex Balance System in healthy adults (mean age 48.9±15.4) showed stronger associations between relatively easy levels of machine-based assessments and manual balance tests [51]. In this study, an assessed stability index on the Biodex Balance System indicating the degree of body movement during the balance test was highly correlated with TUG scores especially at relatively easy Biodex Balance System levels (higher stability of the foot platform). This is comparable to our results including higher correlations between the simple and moderate PTMTs (less digits to connect/ less path of movement) and TUG. Significant stronger associations with relatively easy tasks (simple and moderate PTMT) indicate that the assessment of balance performance using motor-cognitive Physiomat tasks should be conducted at a simple or moderate level.

#### Test–Retest Reliability

Almost all Physiomat outcome measures showed moderate-to-high correlations between test and retest assessment indicating good test–retest reliability. Both temporal (speed of task performance) and spatial (accuracy of task performance) parameters of Physiomat tasks showed similar test–retest reliability. These findings are comparable to results of test–retest analyses of temporal (time used to complete the test) and spatial (the extent of the path moved by the COP during the test) variables of different dynamic balance tasks using a force platform with visual biofeedback in nondemented female nursing home residents [23].

Most previous studies excluded patients with dementia. Exclusion might be based on the assumptions that cognitively impaired persons show an increasing variability of test performance due to illness-related symptoms such as attentional deficits, inability to follow instructions, and impaired executive function. Such dementia-related characteristics challenge an accurate assessment and substantially restrict the reproducibility of specific performances (eg, [52,53]). The only study we found including persons with Alzheimer’s disease showed similar results [33] analyzing test–retest reliability for temporal (reaction time) and spatial (maximum excursion during test performance) variables.

Regarding test–retest reliability of motor-cognitive Physiomat tasks (PTMTs), we found an association between the complexity level and reproducibility. Spearman’s rank correlations and ICCs were lower for simple PTMT compared with moderate and complex motor-cognitive tasks. We could exclude effects of deviating subsamples in different conditions (larger sample in simple condition, selection to high functioning participants in more complex conditions) by subsequent statistical test–retest analyses of 47 participants that successfully conducted all complexity levels. Results of subanalyses confirmed results of the nonselected group as ICCs were lower for simple PTMT compared with moderate and complex motor-cognitive tasks. Referring to this, we suggest a task-specific learning effect from simple to complex PTMTs, which may have led to smaller test performance variability and increased reproducibility regarding complex tasks. Such task-specific learning effects from simple to complex tasks have been reported by Lezak et al [54] attributed to the results of Oliveira et al [55]. The scientists argued that during an initial test, strategies to manage the task might have been developed, which facilitate performing subsequent tasks [54]. Such training mechanisms might have contributed to more reliable test results of complex PTMT in this study, as participants may have felt easier, and more competent in executing the tasks while gaining confidence and stability in performance by prolonged testing.

#### Sensitivity to Change

Sensitivity to change was good to excellent for Physiomat assessment as it reproduced significant improvements regarding all trained Physiomat tasks (FTBT as well as simple, moderate, and complex PTMTs) after a 10-week (twice a week/10 minutes) intervention period. In this study, temporal parameters of PTMTs and FTBT appeared to be more sensitive to change, as effect sizes of test duration (time) were larger than those of accuracy (sway path). Differences might be the result of the test instruction to “perform the FTBT and the PTMTs as fast—not as accurate—as possible,” which focused on speed rather than accuracy of action. Results refer to a “speed-accuracy tradeoff” also reported in an intervention study [56] showing that participants were able to navigate quicker through a test path to measure foot placement but suffered the loss of accuracy after dance video game training. Results may also be influenced by variance of measurement as spatial outcomes showed higher SD compared with temporal outcomes leading to smaller effect sizes.

The complexity level of PTMTs seems to be relevant for responsiveness of Physiomat assessment. Whereas participants showed significant changes with low to high effect sizes in simple and moderate PTMTs, highest effect sizes were found under more challenging conditions (complex PTMT). Results confirmed previous findings from our research group in patients with dementia that more challenging tasks showed higher training gains, in case challenging tasks were still feasible for participants [57]. It is the very large effect sizes documented in highly trained outcomes representing the maximal potential change to be achieved, which are of paramount methodological interest in this study. These large effect sizes indicated the excellent sensitivity to change for the Physiomat assessment. Results supported the task-specific assessment approach as developed for the computerized game-based training and assessment program to document task-specific training gains.

#### Feasibility

Physiomat assessment was feasible even in an old and frail sample with mild-to-moderate dementia. Willingness of the participants to attend in the computerized game-based Physiomat assessment was excellent as only 1 very frail subject, who expressed fear, refused assessment. Results were in line with other reports in force platform–based assessment strategies that are comparable with Physiomat measures, which indicated adequate participation in machine-based computerized tests [58] and a high response rate in patients with dementia [33].

In this present study, all subjects could cope with the FTBT, and most participants could perform lower complexity levels of simple and moderate PBTs and simple and moderate PTMTs. As expected, response rate of the complex tasks was lower based on the higher request on motor-cognitive performance. According to the participants’ reports, fatigue based on motor-functional or cognitive limitations was the primary reason for discontinuation. Unfortunately, based on the participants’ reports, we could not further specify results. Report of fatigue may have been caused by advanced motor impairment and frailty in the study sample or by psychological mechanisms. Previous results of the working group documented that repressive coping strategies or denial of events were significantly associated with inadequate reports on anxiety-related events such as falls in old age [59]. As denial is distinctive of types of dementia [60], we supposed that repressive coping strategy may have led to underreporting of cognitive limitations causing fatigue.

Completion time of Physiomat assessment averaged 25.8 minutes for the comprehensive Physiomat test protocol including a detailed and clear instruction, several trials and breaks between single tasks, and performance levels. As in the original test protocol, more than 3 tasks (simple, moderate, and complex PTMTs) as documented in this validation study were assessed and the completion time to perform those will be substantially reduced. Time to complete ranged from 6 to 55 minutes because of a large heterogeneity of the participants with respect to motor-functional and cognitive status. Completion time of a variety of noninteractive computer-based cognitive tests or batteries to assess or detect age-related changes in cognition ranged from 15 to 60 minutes [61]. Time to complete assessments that is directly comparable to the presented Physiomat measures such as force platform–based assessments [23,33] was not mentioned in the papers.

Participants’ safety was a clear focus in this study as training and assessment was tightly supervised, and clear and brief instructions were provided. As a result, all Physiomat tasks included in the study could be performed safely in this challenging sample of cognitively impaired older adults as no clinical events, falls, and slips could be documented. Safety outcomes are in line with a comparable study examining test–retest reliability of a force platform assessment in people with dementia [33].

### Limitations

Increasing complexity levels in different task conditions led to decreasing sample sizes for each condition. Although we confirmed results of the whole group in the subsample of persons who participated in all test, comparability of psychometric quality may be influenced by slight change of samples between conditions.

### Conclusions

Study results confirm good-to-excellent psychometric quality of an internal assessment approach using quantitative data derived from a computerized game-based training program (Physiomat) in frail persons with mild-to-moderate stage of dementia. This approach provides quantitative parameters that relate to identical and integrated performances trained by using Physiomat and are therefore direct marker of motor-cognitive Physiomat training tasks. Physiomat assessment may represent an evaluation strategy to document game performances and training-associated effects in a rapidly increasing research field including serious games, virtual reality, and machine-based, computerized training.

## Abbreviations

CERAD: Consortium to Establish a Registry for Alzheimer's disease
CI: confidence interval
COP: center of pressure
FES-I: Falls-Efficacy-Scale-International
FTBT: Physiomat Follow-The-Ball Task
GDS: Geriatric Depression Scale
ICC: intraclass correlations
MMSE: Mini-Mental-State Examination
POMA: performance-oriented mobility assessment
PTMT: Physiomat Trail-Making Task
PBT: Physiomat-Balance Task
SD: standard deviation
SRM: standardized response mean
TMT: Trail-Making-Test
TUG: Timed-Up-and-Go
ZN-G: Zahlen-Nachsprechen-G (Repeating-Number-Test)
ZVT-G: Zahlen-Verbindungs-Test-G (Connecting Number-Test)
